# Fermented, Freeze-Dried Snacks from *Lactarius deliciosus* as a Source of Functional Compounds and Lactic Acid Bacteria

**DOI:** 10.3390/molecules30071566

**Published:** 2025-03-31

**Authors:** Kavya Venugopal, Paweł Satora, Katarzyna Kała, Katarzyna Sułkowska-Ziaja, Agnieszka Szewczyk, Beata Ostachowicz, Bożena Muszyńska, Emilia Bernaś

**Affiliations:** 1 Department of Plant Products Technology and Nutrition Hygiene, Faculty of Food Technology, University of Agriculture in Krakow, 122 Balicka, 30–149 Krakow, Poland; kavya.venugopal@student.urk.edu.pl; 2 Department of Fermentation Technology and Microbiology, Faculty of Food Technology, University of Agriculture in Krakow, 122 Balicka, 30–149 Krakow, Poland; 3 Department of Medicinal Plant and Mushroom Biotechnology, Faculty of Pharmacy, Jagiellonian University Medical College, 9 Medyczna, 30–688 Krakow, Poland; k.kala@uj.edu.pl (K.K.); katarzyna.sulkowska-ziaja@uj.edu.pl (K.S.-Z.); agnieszka.szewczyk@uj.edu.pl (A.S.); bozena.muszynska@uj.edu.pl (B.M.); 4 Department of Medical Physics and Biophysics, Faculty of Physics and Applied Computer Science, AGH University of Krakow, 30 Adama Mickiewicza, 30–059 Krakow, Poland; beata.ostachowicz@fis.agh.edu.pl

**Keywords:** wild mushrooms, fermentation, freeze-drying, indoles, ergothioneine, glucans, minerals, antioxidants

## Abstract

*Lactarius deliciosus* is an edible, seasonal, wild-growing forest mushroom with significant functional properties and potential applications in health-promoting foods. The aim of the study was to compare the level of selected functional compounds (minerals, phenols, indoles, L-phenylalanine, lovastatin, ergothioneine, glucans, chitin, chitosan) and Lactic Acid Bacteria (LAB) in freeze-dried snacks made from the fermented caps of *L. deliciosus* mushrooms. The snacks were made from mushrooms blanched in water or microwave, and fermentation was carried out using one of the strains of probiotic bacteria: *L. acidophilus* (LA-5) or *L. plantarum* (SWA016). After 6 months of storage, mushroom products were a good source of functional compounds, especially LAB, minerals, indoles, lovastatin, antioxidants (phenolic compounds), and dietary fibre. Fermentation with added probiotic cultures enhanced indigenous lactobacilli levels, but after storage, only microwave-blanched snacks fermented with *L. plantarum* retained a high LAB count (7.3 log CFU/g). The selection of pre-treatment significantly influenced bioactive compound composition: water blanching enhanced lovastatin and 6-methyl-D,L-tryptophan contents, whereas microwave blanching maximised K, S, Rb, Fe, Se, Mn, Br, phenolic compounds, antioxidant activity, and soluble dietary fibre. In order to optimise the level of the most important bioactive compounds and LAB, microwave blanching with the addition of *L. plantarum* SWA016 should applied.

## 1. Introduction

In recent years, there has been an increase in consumer awareness about the influence of diet on health, which has translated into a growing interest in functional products, which can include mushrooms. Ready-to-eat snacks with health-promoting substances are one of the most rapidly growing directions in food processing. Fruiting bodies of edible mushrooms, especially wild species, can be a good raw material for the snack industry due to their extraordinary organoleptic and health-promoting qualities.

*Lactarius deliciosus* (L.) Pers. of the family *Russulaceae* is valued not only for its culinary qualities but also for its rich array of bioactive compounds with potential therapeutic benefits. This mushroom contains a variety of secondary metabolites, including organic acids, alkaloids, terpenoids, steroids, and antioxidant compounds such as phenolic compounds, polyphenols, and phenylpropanoid glycosides such as lactaroviolin, which is unique to *L. deliciosus* species [1]. Its nutritional profile includes carbohydrates, proteins, low fat and free sugars, as well as mannitol, a six-carbon sugar alcohol, stearic acid as the primary fatty acid, and quininic acid as the dominant organic acid. In addition, *L. deliciosus* is rich in phenolic compounds, including p-hydroxybenzoic acid, and contains around twenty free amino acids and various volatile compounds, mainly aldehydes. Mushrooms are effective in accumulating macro- and microelements such as potassium, phosphorus, sodium, calcium, and trace elements, such as copper and zinc, which are essential for cellular functions [1,2,3,4,5,6].

The cell wall of *L. deliciosus* is mainly composed of polysaccharides, including chitin and various glucans, which are increasingly recognised for their biological activity. These polysaccharides act as biological response modulators with potential immunostimulatory, antioxidant, and antitumor properties. Recent research has identified a polysaccharide from *L. deliciosus* (LDG–A), which contains L-mannose and D-xylose in a 3:1 ratio and shows significant potential for antitumour and immunomodulatory effects by enhancing immune cell proliferation, T and B cell activation, and lymphokine secretion [7]. Although the precise mechanisms by which fungal polysaccharides exert their antitumour effects are still under investigation, their role in modulating host immune responses is well documented [8]. *L. deliciosus* extracts exhibit remarkable antimicrobial activity against bacteria and fungi, a property that correlates with the presence of phenols, flavonoids, ascorbic acid, β-carotene, and lycopene. However, these compounds decrease as the mushroom matures and during storage [2,9,10,11,12,13].

Various preservation methods, including traditional techniques such as drying and pickling and novel methods such as freezing, UV irradiation, and microwave drying, affect the quality and nutritional retention of the mushrooms. Lacto-fermentation, an ancient preservation method, is particularly promising for enhancing the bioactive compounds in mushrooms, improving their nutritional value and contributing to their umami flavour [14,15]. As shown by Jabłońska et al. [16] and previous studies [17], blanching is usually an essential step during the fermentation of mushrooms, having a positive effect on their quality, especially texture and colour. Blanching helps enzyme inactivation and in enhancing long-term storage quality. This pre-treatment also increases protein digestibility and induces Maillard reactions, which contribute to specific sensory characteristics [12,18,19]. Water blanching is a traditional method of pre-treatment before mushroom fermentation, but this treatment can cause water-soluble components, such as B vitamins, glucans, minerals, etc., to leach from the tissue. Considering the economic viability and sustainability, microwave blanching can be a good alternative because it reduces nutrient losses. Therefore, understanding how different pre-treatment methods affect the nutritional and bioactive properties of lacto-fermented *L. deliciosus* requires careful control and monitoring of fermentation parameters.

Microbes involved in fermentation release bioactive peptides from proteins, as well as a wide variety of metabolites, including short-chain fatty acids (SCFAs), vitamins, exopolysaccharides, conjugated linoleic acid (CLA), and γ-aminobutyric acid, which play an important role in improving health and also contribute to the sensory characteristics of fermented foods [20]. The symbiotic relationship between fermented foods and the human gut microbiome underscores their potential to promote gastrointestinal, metabolic, immune and mental health. Psychobiotics, an emerging field exploring the therapeutic potential of fermented foods, represents a promising avenue for further investigation [21]. As research in this area advances, it promises to shed light on the complex interplay between dietary interventions and human health outcomes, opening avenues for innovative therapeutic strategies and personalised nutritional approaches [22,23,24,25].

The aim of this study was to compare the health-promoting properties of mushroom snacks made from the fermented caps of *L. deliciosus* mushrooms. This research is a continuation of the already published first-stage studies [17] and constitutes the second stage of this research. In the first stage, the aim was to select an optimal pre-treatment method (water or microwave blanching) before fermentation that would facilitate a rapid decrease in the product pH (< 4.5) while ensuring favourable organoleptic quality and high nutritional value. Three blanching methods were tested: standard blanching in water for 120 s, microwave blanching for 120 s, and short blanching in water for 30 s. Based on the results, an optimal pre-treatment was identified for each added probiotic strain. For *Lactobacillus acidophilus* strain LA-5, the best pre-treatment was blanching in water for 30 s, and for *Lactiplantibacillus plantarum* strain SWA016, microwave blanching for 120 s. The second stage of the study aimed to evaluate and compare the functional and probiotic properties of fermented and freeze-dried *L. deliciosus* mushroom snacks after 6 months of storage. Before fermentation, the mushrooms were blanched in water or microwave, and fermentation was carried out using one of the probiotic strains (*L. acidophilus* (LA-5) or *L. plantarum* (SWA016)). The study investigated the effects of two parameters: the blanching method and the addition of probiotic bacteria.

## 2. Results and Discussion

### 2.1. Lactic Acid Bacteria Content in Fermented and Freeze-Dried Mushrooms After 6 Months Storage

*L. deliciosus* mushrooms were fermented, freeze-dried, and analysed for two groups of LAB after fermentation and again after 6 months of storage at 4 °C. After fermentation, lactococci predominated in fermented mushrooms without added probiotic cultures (Table 1). Water blanching effectively eliminated indigenous strains of lactobacilli, thereby reducing the microbial load post-fermentation, while microwave blanching was less effective, likely due to non-uniform heating. These findings align with the authors’ previous research on the *Agaricus bisporus* mushroom [26]. The addition of probiotic cultures *L. acidophilus* or *L. plantarum* significantly increased the levels of indigenous lactobacilli post-fermentation, with counts exceeding 8 log CFU/g, which is well above the probiotic threshold of 10^6^ CFU/g required for probiotic classification [27]. The freeze-drying process and subsequent storage had a significant effect on the LAB content of the products. The levels of both lactococci and lactobacilli were reduced by at least one log in all samples regardless of the addition of probiotics during the fermentation. The reduction in the number of live bacterial cells was evident in water-blanched samples with added *L. acidophilus*, where no probiotic cells were detected after freeze-drying and storage, suggesting a heightened sensitivity of this strain to the combined effects of blanching, dehydration, and prolonged storage. The only product in which the presence of Lactobacilli was noted was microwaved mushrooms (MB + LbP). As shown by Yang et al. [28], during storage of fermented juices, LAB abundance decreased dramatically after 20 days, with slightly higher viability among the different *L. plantarum* strains. It is difficult to conclude unequivocally what caused such a phenomenon; nevertheless, it can be assumed that the microwave treatment resulted in lyoprotectants in the product, which may have stabilised the *L. plantarum* during storage but did not stabilise other types of LAB [29]. There are currently no reports on the antimicrobial effect of *L. deliciosus* constituents on LAB. However, *L. deliciosus* contains polyacetylenes, compounds known for their bactericidal activity [30]. It can be hypothesised that the freeze-drying process concentrates these substances by dehydration, and certain LAB cultures, i.e., *L. acidophilus* may be particularly sensitive to their effects. While this presents a challenge for probiotic stability, it also highlights a unique aspect of *L. deliciosus* composition that may contribute to its natural preservation potential.

Compared to previous studies on fermented probiotic foods, this research shows that freeze-dried fermented mushrooms can be an attractive alternative to standard fruit, vegetable, and meat snacks rich in Lactococci bacteria and functional compounds like glucans. However, a key limitation is the significant loss of Lactobacilli after freeze-drying and storage, which raises concerns about long-term probiotic stability. This highlights the need for optimisation strategies, such as encapsulation or co-fermentation with protective adjuncts, to improve bacterial survival in future studies. Despite this limitation, the study demonstrates the feasibility of mushroom-based probiotic products and expands knowledge on how different processing methods impact microbial stability in novel fermentation substrates.

### 2.2. Water Activity

Water activity is a key factor in the microbiological and physicochemical stability of freeze-dried mushrooms [31]. Fresh mushrooms typically have a water activity >0.950, higher than most vegetables and meats [32]. After freeze-drying, *L. deliciosus* samples had water activity below 0.270 (Table 2), indicating effective moisture removal. Microwave-blanched samples had the highest water activity (0.26–0.27), followed by non-blanched (0.24) and water-blanched (0.22–0.24). Water blanching likely enhanced protein denaturation and leaching of water-soluble compounds, reducing water retention [33]. In contrast, microwave blanching may have led to uneven moisture distribution, contributing to slightly higher water activity.

### 2.3. Organic Compounds

Mushrooms are a rich source of bioactive compounds, including lovastatin, γ-aminobutyric acid (GABA), and ergothioneine, which have been linked to cholesterol-lowering and antioxidant effects. Additionally, mushrooms contain indole derivatives such as L-tryptophan, 5-hydroxy-L-tryptophan, 6-methyl-D,L-tryptophan, tryptamine, 5-methyltryptamine, serotonin, and melatonin, all of which play key roles in brain chemistry, affecting mood regulation, sleep, and neurotransmission. While some of these compounds are relatively heat-stable, others are more sensitive to thermal processing, which can diminish the therapeutic potential of these bioactive compounds due to heat-induced stress and oxidation [34,35,36,37,38,39]. According to Chen et al. [34], of the various mushroom species, *Cordyceps sinensis* and *Agaricus blazei* are the most abundant in lovastotin and γ-aminobutyric acid (76.9–136.5 mg/100 g dm and 20.0–22.0 mg/100 g dm, respectively), while ergothioneine is found in *Pleurotus ostreatus* and *Pleurotus citrinopileatus* (145.8–285.0 mg/100 g dm). Against these species, *L. deliciosus* products contained fewer amounts of lovastotin and, in many cases, a greater of ergothioneine (Table 2). In this study, non-blanched samples exhibited the highest concentrations of ergothioneine, L-tryptophan, 5-HTP, and p-hydroxybenzoic acid, suggesting that these compounds are more stable in their natural state. Water blanching significantly increased lovastatin and 6-methyl-D,L-tryptophan, but also caused a significant reduction in 5-HTP, highlighting the dual nature of thermal processing where some bioactive compounds are concentrated and others are diminished due to thermal degradation or leaching. Microwave blanching increased 5-HTP and 5-methyltryptamine levels by 95% and 37%, respectively, suggesting that microwave heating may enhance the extract or synthesis of these compounds more efficiently than water blanching which underscores the importance of precise control over heating techniques to optimise the bioactive profile in fermented mushrooms (Table 2). *P. ostreatus* is considered the best source of lovastatin (60.6 mg/100 g dm), while *P. djamor* 5-hydroxy-L-tryptophan (5-HTP) (193.9 mg/100 g dm) [34,35,36,37,38,39].

Interestingly, probiotic fermentation led to lower levels of ergothioneine, 5-methyltryptamine, lovastatin, and L-tryptophan, likely due to microbial metabolism altering the compound profile. This suggests that fermentation with specific bacterial strains can modify the bioactive components, possibly through degradation or transformation into other metabolites. These findings align with those of Paramithiotis et al. [40], who reported that bacterial composition plays a crucial role in shaping the bioactive profile of fermented products. This emphasises the need for careful selection of bacterial strains to achieve the desired therapeutic properties in fermented mushroom products. In conclusion, while thermal and fermentation processes can enhance or concentrate certain bioactive compounds, they also degrade others. These findings show the complexity of mushroom processing and the need for optimised methods that preserve both the nutritional and therapeutic potential of mushrooms [36,37].

### 2.4. Mineral Substances

*L. deliciosus* mushrooms are a good source of mineral substances, especially K, P, Mg, Fe, Na, Mn, Ca, and Zn [3,4]. One of the most effective ways to enhance the mineral composition of foods is fermentation. A study by López et al. [3] showed that *L. deliciosus*, which is abundant in minerals, particularly K, P, Mg, Fe, Na, Mn, Ca, and Zn [3,4], contained approximately 5–10 times higher levels of K and Ca after fermentation, highlighting the potential of fermentation as a strategy for enhancing mineral bioavailability in mushrooms. As shown by Lee et al. [41], different processing methods (cooking, blanching, steaming, roasting, boiling, microwaving) affected mineral and B vitamin levels in shiitake mushrooms. The greatest losses were from cooking, and the smallest losses were caused by roasting and microwaving. Potassium level was determined in the highest amount, next P, Mg and Ca. In this study, non-blanched mushrooms exhibited the highest mineral concentrations in the order: K > Ca > S > Cl > Ga > Zn > Rb > Fe > Cu > Ni > Sr > Se > Mn > Br > Cr (Table 3), providing a baseline for comparison with processed products. However, microwave-blanched mushroom samples, including those with probiotic bacteria (MB, MB + LbP), displayed higher contents of K, S, Rb, Fe, Se, Mn, and Br, likely due to the evaporation of a small amount of water from the tissue during treatment, potentially leading to increased mineral concentration in the remaining mushroom tissue. This indicates that microwave blanching may help preserve or even concentrate certain minerals, but this effect should be considered in relation to the potential loss of other nutrients during the process. Water-blanched samples contained the lowest levels of most minerals, very likely due to leaching into the blanching water, which predominantly affects the water-soluble minerals like K, Cl and Br [42,43]. The effect of probiotic bacteria on mineral content is more complex and influenced by their growth dynamics. Probiotic bacteria can release minerals from the mushroom substrate through protein and carbohydrate breakdown, increasing their bioavailability. These minerals may leach into the brine or be utilised by the bacteria for their growth [44]. The interactions between probiotic bacteria and the mineral composition should be further explored to understand the nutritional impact on fermented mushroom products.

### 2.5. Antioxidant Activity and Phenols Profile

Phenolic compounds, including flavonoids, anthocyanins, phenylpropanoids, and carotenoids, are known for their redox properties, acting as reducing agents, hydrogen donors, and singlet oxygen quenchers. These properties directly correlate with the antioxidant activity in mushrooms [10,45,46,47,48]. However, to accurately assess antioxidant capacity, multiple measurement methods are necessary to account for the different mechanisms through which antioxidants act [49]. It is well-established that phenolic compounds are heat-sensitive and water-soluble, making them prone to degradation during blanching or storage, depending on oxygen availability and light exposure [50]. This highlights the importance of optimising processing methods to minimise the loss of these valuable compounds while preserving their antioxidant properties.

In this study, the total phenolic content ranged from 1815 to 2663 mg/100 g dm, with the highest amounts observed in microwave-blanched samples (MB, MB + LbP) and the lowest in water-blanched samples (Table 4). These findings align with literature data [51], which report *L. deliciosus* to contain 239–26,370 mg GAE/100 g dm of total phenolics, depending on harvest location and extraction method. The addition of probiotic bacteria consistently increased phenolic content by 5–33%, irrespective of the blanching method. This suggests that probiotic fermentation plays a significant role in enhancing the antioxidant capacity of mushrooms, likely by promoting the release or synthesis of phenolic compounds through microbial metabolism. Lycopene and beta-carotene contents ranged from 2.00–4.11 mg and 2.58–8.65 mg/100 g dm, respectively, which were consistent with results obtained for fresh *L. deliciosus* by Barros et al. [10] and Sharma et al. [52]. The highest levels of lycopene and β-carotene were found in freeze-dried fresh and non-blanched mushrooms, indicating that both blanching methods result in a reduction of these compounds. Water-blanched products contained higher lycopene and β-carotene levels than microwave-blanched products, suggesting that microwave heating may cause greater degradation of these carotenoids due to its intense energy input. However, a different trend was observed in fermented products with probiotic strains, suggesting that fermentation may alter the stability of these carotenoids in unexpected ways.

Processing methods have varying effects on the antioxidant activity in mushrooms. Pre-treatments, such as steam or microwave blanching, are known to retain better antioxidant activity than water blanching, as they prevent phenolic compound loss due to leaching [53,54]. In this study, microwave-blanched products (MB, MB + LbP) generally exhibited the highest antioxidant activity, while water-blanched samples had the lowest. Furthermore, for microwave-blanched mushrooms, spontaneous fermentation was more beneficial than controlled fermentation, likely due to the natural metabolic processes of the microbes involved, which might be more effective at enhancing antioxidant activity. Conversely, for water-blanched mushrooms, fermentation with probiotic bacteria significantly improved antioxidant activity, supporting findings by Mousavi et al. [55], who demonstrated that fruit juices fermented with *L. acidophilus* had higher antioxidant activity compared to those fermented with *L. plantarum*.

### 2.6. Dietary Fibre and Polysaccharides

Dietary fibre in mushrooms consists of non-starch polysaccharides, primarily chitin, lignin, α– and β–glucans, cellulose, and hemicelluloses (e.g., mannans, xylans, and galactans), which pass through the small intestine undisturbed. Upon reaching the colon, they are fermented by intestinal bacteria. While soluble dietary fibre (SDF) was previously considered more functional than insoluble dietary fibre (IDF), recent studies highlight the hypolipidemic effects of IDF and its role in producing beneficial short-chain fatty acids through gut microbial activity. The majority of β-glucans are resistant to gastric juice and pass through the small intestine unaltered, where they attach to immune cells and stimulate an anti-cancer response [56,57,58]. The reference daily intake (RDI) for total dietary fibre (TDF) is 25–30 g for adults, and according to Xu et al. [7], fresh *L. deliciosus* mushrooms contain 31.81 g of TDF, 26.51 g of IDF, and 5.30 g of SDF/100 g dm, exceeding dietary recommendations. This indicated that *L. deliciosus* mushrooms are a significant source of dietary fibre. In this study, the contents of TDF and SDF were higher in fermented and blanched samples compared to non-fermented and non-blanched samples (Table 4). This suggests that fermentation and blanching can enhance the fibre profile, potentially increasing the nutritional value of mushrooms, which could have broader health benefits. The highest TDF content (50.38–51.83 g/100 g dm) was observed in water-blanched products, likely due to dietary fibre depolymerisation caused by heat treatment [59,60]. This finding suggests that heat treatment may break down the fibrous matrix, resulting in higher concentrations of dietary fibre but also possibly affecting the structural integrity of certain fibre types. This highlights the complex relationship between processing methods and fibre retention, with water blanching potentially concentrating some types of fibre while altering others. Samples with added probiotic bacteria generally exhibited higher levels of TDF in both pre-treatments, indicating that probiotics may enhance dietary fibre content, possibly through microbial metabolism or fermentation processes. However, further research is needed to understand the mechanisms by which probiotics influence fibre levels and their impact on the final product. Approximately 10% of the TDF in all samples was SDF, with h levels slightly higher in microwave-blanched samples than in water-blanched samples. The use of microwave blanching likely prevented the leaching of the soluble compounds, compared to water blanching, which may have aided in SDF preservation more effectively. The effect of fermentation on dietary fibre is complex. On the one hand, LAB utilises fibre for growth, reducing its content in the product. On the other hand, their enzymatic activity can convert monomers into polymers or even depolymerise dietary fibre [61,62,63,64]. On the other hand, fermentation can also cause an increase in TDF and SDF, as they showed Zhao et al. [65] during a study of fermented wheat bran. This dual effect of LAB highlights the adaptive nature of microbial metabolism, which can both degrade and synthesise fibre components depending on the conditions and microbial strains used, which is also observed in other plant product fermentation, which can be applied in mushroom fermentation, suggesting that certain fermentation conditions or strains could similarly enhance fibre content in mushrooms during fermentation.

In the study by Mirończuk–Chodakowska and Witkowska [56], the total, α- and β-glucan content in fresh *L. deliciosus* were lower by 0.89 g, 2.35 g, and 3.29 g, respectively, compared to the values presented in Table 4. These discrepancies could be attributed to differences in mushroom variety, harvest conditions, or methodological variations between studies. In both fermented and unfermented mushrooms, β-glucans predominated, comprising 96.0–97.5% of total glucans, indicating their dominance in the glucan profile of *L. deliciosus*. This is consistent with previous findings, where β-glucans are often the most abundant type of glucan in edible mushrooms. Total glucans were highest in non-blanched samples and lower in microwave-blanched products, suggesting that microwave treatment may result in some glucan loss, likely due to heat-induced degradation or structural changes. Interestingly, products fermented with the addition of probiotic bacteria (WB + LbA, MB + LbP) contained 11–27% higher levels of total and β-glucans than those fermented without added probiotics. This indicates that probiotic bacteria not only play a role in enhancing fermentation processes but also in increasing the synthesis or release of β-glucans, which aligns with the findings of Bibi et al. [66] and Di Cagno et al. [67], who demonstrated that LAB species can synthesise exopolysaccharides, including β-glucans.

The chitin and chitosan content in fermented *L. delicious* was 20–23 g and 0.15–0.64 g/100 g dm, respectively, depending on the pre-treatment type and probiotic bacteria addition (Table 5). For comparison, chitin content in *Ganoderma lucidum* ranges from 34–40 g, while in *Agaricus bisporus,* 7–10 g [68,69]. This suggests that *L. deliciosus* contains a significant amount of chitin, which plays a role in the structural integrity of the fungal cell wall. Fermentation led to an increase in chitin content by 1.40–3.10 g, suggesting that microbial activity may enhance chitin synthesis or prevent its degradation. However, chitosan levels decreased by 0.23–0.49 g, likely due to partial dissolution in the acidic brine environment during fermentation, which does not affect chitin [70]. Interestingly, non-blanched samples exhibited the lowest chitin content but the highest chitosan levels, indicating that processing methods can influence the conversion between these two compounds. Conversely, microwave-blanched products contained more chitosan than water-blanched samples, suggesting that microwave treatment may preserve chitosan by preventing its dissolution in the brine or possibly enhancing its formation during microwave processing.

## 3. Materials and Methods

### 3.1. Mushroom Preparation

Fresh caps of *Lactarius deliciosus* mushroom (diameter 4–8 cm) were purchased from the local market in Kraków (Poland), cleaned with tap water (15 °C), and processed. The caps measuring 6–8 cm in diameter were cut in half. The taxonomic identification was carried out by an expert authorised by the Provincial Sanitary Inspector of Małopolska (Poland) using the software MycoKey 4.1. All of the mushrooms were of a similar maturity. Representative voucher specimens were deposited at the Department of Plant Products Technology and Nutrition Hygiene, University of Agriculture in Kraków, Poland. The mushroom caps were fermented without blanching (control) or after blanching in water for 30 s (temp. 96–98 °C; ratio 1:5 mushrooms: water, *w*/*w*) and in the microwave (2 min, 1000 W, 2.45 GHz). After water blanching, the caps were cooled in cold tap water and placed on sieves to remove excess water. Microwave blanching was performed in a Panasonic NN-F621MB EPG microwave oven. Portions of 500 g of mushrooms were placed in a glass bowl in the microwave and mixed twice during blanching to ensure even heating. After microwave blanching, the material was spread out on trays in a layer of approximately 1–2 cm to reduce the temperature of the fruiting bodies to below 25 °C.

### 3.2. Preparation of Probiotic Bacteria

Bacterial cultures were stored in lyophilised form. A specific amount of each probiotic bacterial strain was rehydrated in 10 mL of MRS broth. The entire culture was incubated in a CO_2_ incubator at 37 °C with 15% CO_2_. After 48 h, the culture was transferred to a further 10 mL of MRS medium. Fed-batch cultures were continued by adding a fresh portion of medium every 24 h until the final volume of bacterial growth reached 100 mL. Samples were vortexed daily at a speed of 2000 rpm until the cell pellet was completely suspended in the medium. After one week of cultivation, the bacterial density was determined by measuring the optical density using a McFarland scale. The volume of the bacterial slurry to be added to the mushrooms was calculated to achieve an initial concentration of 10^10^ cells/kg of fruiting bodies. The calculated volume was centrifuged at 5000 rpm for 10 min at 4 °C (MPW-365 centrifuge, Warsaw, Poland) and resuspended in sterile Ringer’s solution. LbA and LbP were added separately to the brine and stirred.

### 3.3. Fermentation Process, Freeze-Drying, and Storage

After the blanching process, the caps were placed in fermentation vessels, covered with a brine solution of 2% NaCl and 3% sucrose (spontaneous fermentation) or 2% NaCl and 3% sucrose with the addition of one of the strains of probiotic bacterial strains: *Lactobacillus acidophilus* strain LA5 (Hansen, code LbA) or *Lactiplantibacillus plantarum* strain SWA016 (obtained from Swanson, code LbP) (controlled fermentation). The ratio of brine to mushroom caps was 1:2 (*w*/*w*). The mushroom caps were loaded with sinkers, placed in the incubator and fermented at 26 ± 1 °C for 3 days until pH ≤ 4.5. Next, the products were transferred to a refrigerator and stored at 5 ± 1 °C for the next 14 days (total fermentation: 17 days) to stabilise and allow the development of flavour compounds. The sinkers were cleaned daily by washing them thoroughly under cold and running tap water. At the end of the fermentation process, the products were placed on sieves and left for 30 min at room temperature to separate the mushroom caps from the brine. Fresh and fermented mushroom caps were frozen in a single layer (approx. 2 cm thick) by the air-blast method at −40 °C for 120 min to reach −25 °C in the thermal centre of the sample. After freezing, all mushroom samples (fresh and fermented) were freeze-dried using a Gamma 1–16 LSC freeze dryer (Martin Christ Gefriertrocknungsanlagen GmbH, Osterode am Harz, Germany) with the following parameters: initial product temperature: −30 °C; condenser temperature: −52 °C; shelf temperature: 20 °C; and for secondary drying (last 6 h of total drying time): shelf temperature: 25 °C. Drying was carried out for approximately 48 h to a moisture content of less than 3%. The dried mushrooms were packed in plastic bags designed for vacuum packaging (vacuum 70%) (Vacuum System Boxer 42, Henkelman B.V., CK’s-Hertogenbosch, The Netherlands) and stored in the absence of light for 6 months at 4 °C. The resulting products were called “snacks” because they had the form of a snack and were ready to eat.

### 3.4. Water Activity Analysis

The water activity of the freeze-dried samples (NB, WB, WB + LbA, MB, MB + LbP) was measured in an AquaLab Series 4TE apparatus (Decagon Devices, Pullman, WA, USA), which operates on the basis of dew point detection, according to the specifications provided by the manufacturer. Samples were cut into small pieces (1–2 mm) and placed in a plastic vessel (ø = 40 mm, h = 12 mm) suitable for this measurement, then placed in an airtight measuring chamber of the apparatus. The measurement was carried out at 25 °C.

### 3.5. Microbiological Analysis

At the end of fermentation (before freeze-drying) and after 6 months storage at 4 °C (freeze-dried), the samples (WB, WB + LbA, MB, MB + LbP) were subjected to microbiological analysis to assess the presence of lactic acid bacteria, including the addition of probiotic cultures in accordance to Satora et al. [71]. One gram of fermented mushroom or lyophilisate was added to 100 mL of sterile Ringer’s solution and placed in a stomacher bag. Samples were homogenised for 5 min. Appropriate decimal dilutions were then prepared using tubes containing sterile buffered peptone water (pH 7.0, Biomaxima, Warsaw, Poland). Inoculations were performed using M17 agar (Biocorp, Warsaw, Poland) for the analysis of lactic acid cocci and MRS agar (Bicorp, Warsaw, Poland) for the analysis of lactic acid bacteria. Samples inoculated with M17 medium were incubated at 28 °C, and those with MRS medium were incubated at 37 °C for 72 h in an incubator (17% CO_2_, Esco CCL-170B-8). Only plates with 30–300 colonies were analysed. Colonies were randomly selected, and the microorganisms were analysed microscopically.

### 3.6. Determination of Organic Compounds and Antioxidant Activity

All analyses were carried out on methanolic extracts prepared according to Section 3.6.1.

#### 3.6.1. Preparation of Methanolic Extracts

The methanol extracts were prepared according to the method described by Rząsa-Duran et al. [72]. The freeze-dried samples (NB, WB, WB + LbA, MB, MB + LbP) were ground to a uniform consistency using an agate mortar. To each 4 g sample, 125 mL of analytical-grade methanol was added, and the mixture was placed in an ultrasonic bath (Polsonic, Warsaw, Poland) for 20 min at room temperature (23 ± 2 °C). After sonification, the samples were filtered through filter paper; the filtrates were collected, while residues were left at room temperature to allow methanol evaporation. Once dried, residues were transferred to a beaker, and another portion of methanol (125 mL) was added. The mixture was placed in the ultrasonic bath for extraction under the same conditions as described before. The extraction process was repeated nine times. After extraction, all filtrates were combined and placed in a crystalliser for methanol evaporation (23 ± 2 °C). After methanol evaporation, all filtrates were combined, and the resulting dry residue was dissolved in HPLC-grade methanol. The solution was then filtered using 0.22 µm PTFE syringe filters (ChemLand, Stargard, Poland), and each sample was analysed in triplicate.

#### 3.6.2. Analysis of L-Phenylalanine and p–Hydroxybenzoic Acid

The RP-HPLC-DAD method was used to determine the content of L-phenylalanine and p-hydroxybenzoic acid [73]. The HPLC VWR/Hitachi LaChrom Elite analyser (Merck Hitachi, Tokyo, Japan) was equipped with a DAD-L2455 detector. The LiChrosfer RP-18 column (4 × 250 mm, 5 µm) was used. The mobile phase consisted of two solvents: A (methanol and 0.5% acetic acid solution, ratio of 1:4, *v*/*v*) and B (HPLC-grade methanol). The analysis was carried out at 254 nm; the flow rate was 1 mL/min. Gradient elution was performed as follows: 0–25 min A/B 100/0, 25–35 min A/B 70/30, 35–45 min A/B 50/50, 50–55 min A/B 0/100 and 57–67 min A/B 100/0 (*v*/*v*).

#### 3.6.3. Analysis of Lovastatin, Ergothioneine, and Indole Compounds

The amount of lovastatin [74], ergothioneine [75], and indole compounds (L–tryptophan, 6-methyl-D,L-tryptophan, 5-hydroxy-L-tryptophan, serotonin) [76] was determined by the RP-HPLC-UV method with some modifications. The HPLC VWR/Hitachi LaChrom Elite analyser (Merck Hitachi, Tokyo, Japan) equipped with a UV detector L-7400 was used for analysis. The analysis was carried out on a Purospher RP-18 column (4 × 250 mm, 5 µm). For lovastatin, isocratic elution was used. The mobile phase consisted of a mixture of acetonitrile and 0.1% phosphoric acid solution in a ratio of 60/40 (*v*/*v*). The flow rate was 1 mL/min, and the detection wavelength was 238 nm. For indole compounds, isocratic elution was also used. For the determination of 5-hydroxy-L-tryptophan, a mixture of 0.1% phosphoric acid and acetonitrile in a volume ratio of 97/3 was used as eluent. The analysis was performed at 280 nm with a flow rate of 1.0 mL/min. The L-tryptophan, 6-methyl-D,L-tryptophan and 5-methyltryptamine contents were analysed with a mixture of methanol, water, and 0.1 M ammonium acetate in a volume ratio of 15/14/1. The flow rate was set at 1.0 mL/min, and measurements were performed at 275 nm. For ergothioneine, two solvents (A and B) were used for the mobile phase. Solvent A was a mixture of water and methanol in a ratio of 99/1 (*v*/*v*) acidified with boric acid to pH = 5.0, while solvent B was HPLC-grade methanol. Gradient elution was performed as follows: 0–7 min: A/B 100/0, 7–10 min: A/B 70/30, 10–14 min: A/B 10/90, 14–19 min: A/B 0/100, 19–21 min: A/B 40/60, 21–23 min: A/B 70/30 and 23–37 min: A/B 100/0 (*v*/*v*). The flow rate was set at 0.5 mL/min, and the detection wavelength was 257 nm.

#### 3.6.4. Determination of Antioxidant Activity (DPPH, ABTS, FRAP)

The antioxidant activity was determined using the DPPH [77], ABTS [78], and FRAP [79] methods. The incubation time was 30 min (DPPH) or 120 min (ABTS); the absorbance was measured at 517 nm (DPPH) or 734 nm (ABTS). The antioxidant activity was expressed in mg of Trolox equivalents (TE) per 1 g of dry matter. For the FRAP method, the absorbance was measured at 593 nm, and the results were expressed as mM Fe^2+^ per 1 g of dm. All samples were incubated in the dark. For all analyses, a UV/VIS Helios Beta spectrophotometer (Thermo Fisher Scientific Inc., Waltham, MA, USA) was used.

### 3.7. Determination of Phenols Profile

The phenol profile (total phenolics, total tartaric esters, total flavonoids, total anthocyanins) was determined using Fukumoto and Mazza [80] method in samples NB, WB, WB + LbA, MB, MB + LbP. The contents of all compounds were determined in 80% methanol extracts (80 mg sample/mL extract) obtained by one-fold homogenisation (90 s) at room temperature (20 ± 2 °C). After homogenisation, the samples were centrifuged (20 min, 5000 rpm). The total phenol, total phenylpropanoids, total flavonoids, and total anthocyanins were measured at 280, 320, 360, and 520 nm on a Shimadzu UV-160 UV-VIS spectrophotometer (Tokyo, Japan). Chlorogenic acid, caffeic acid, quercetin, and cyanidin chloride were used as standards.

### 3.8. Determination of Carotenoids

The carotenoid content in samples NB, WB, WB + LbA, MB, MB + LbP was determined using Nagata and Yamashita [81] method in acetone–hexane (4:6) solution by one-fold homogenisation (60 s) at room temperature (20 ± 2 °C) (0.2 g sample/mL extract). After centrifugation (10 min, 5000 rpm), the optical density of the supernatant was measured at 663, 505, and 453 nm using a Shimadzu UV-160 UV-VIS spectrophotometer (Tokyo, Japan). The carotenoid content was calculated using the following equations:

Lycopene (mg/100 mL) = −0.0458A_663_ + 0.372A_505_ – 0.0806A_453_

β–carotene (mg/100 mL) = 0.216A_663_ – 0.304A_505_ + 0.452A_453_

### 3.9. Determination of Mineral Components

The mushroom samples (NB, WB, WB + LbA, MB, MB + LbP) were homogenised in an agate mortar and mineralised in a microwave system in the Magnum II microwave apparatus (Ertec, Wrocław, Poland) [72]. Approximately 0.2 g of the dried sample was taken and transferred to a Teflon vessel, followed by the addition of 6 mL of 65% HNO_3_ (Suprapure^®^) and 4 mL of 30% H_2_O_2_ (Suprapure^®^). The mushroom material was subjected to a three-stage mineralisation process (295 °C) at 70% and 100% power, respectively. The mineralised mushroom samples were transferred to quartz evaporators, and excess reagents were evaporated at 60 °C for 45 min. The residues obtained were quantitatively transferred to 5 mL volumetric flasks and mineralised in three repetitions. The elemental composition was determined using a TXRF Nanohunter II spectrometer (Rigaku, Wroclaw, Poland) equipped with an X-ray tube containing a molybdenum anode. Measurements were performed for 1000 s at 50 kV power. To analyse the composition of minerals such as K, Ca, S, Cl, Ga, Zn, Rb, Fe, Cu, Ni, Sr, Se, Mn, Br, and Cr, gallium (1000 ppm) was used as an internal standard in the prepared test samples.

### 3.10. Determination of Polysaccharides

The determination of total dietary fibre (TDF), soluble dietary fibre (SDF), insoluble dietary fibre (IDF), and glucans (total glucans, 1,3:1,6-β-glucans and α-glucans) was performed using assay kits from Megazyme Ltd. (Bray, Ireland) in accordance with the manufacturer’s instructions. Measurement of glucans was analysed using the Yeast and Mushroom Assay Kit in samples NB, WB, WB + LbA, MB, MB + LbP.

Crude chitin and chitosan contents were determined according to Synowiecki and Al-Khateeb’s [82] method in samples NB, WB, WB + LbA, MB, MB + LbP. The determination involved deproteinisation of the samples (2% NaOH, 90 °C, 2 h), separation of the alkali-insoluble fraction (AIF) by centrifugation, extraction of chitosan from AIF under reflux (10% acetic acid, 40: l, 60 °C, 6 h), separation of crude chitin by centrifugation, and precipitation of chitosan from the extract at pH 9.0. Samples were washed on a coarse sintered glass funnel (G-4) with water, ethanol, and acetone and, finally, air-dried at 20 °C to maintain constant weight.

### 3.11. Statistical Analysis

Samples were analysed in three or four independent replicates. Results were statistically analysed using one-way analysis of variance (ANOVA) based on Duncan’s range test (*p* < 0.05). Analyses were performed using Tibco Statistic version 13.3 Pl (Stat-Soft, Tulsa, OK, USA).

## 4. Conclusions

The freeze-dried snacks made from fermented *L. deliciosus* mushrooms could serve as a good source of functional compounds, particularly lactic acid bacteria, minerals, 5-methyltryptamine, lovastatin, 6-methyl-D,L-tryptophan, antioxidants (phenolic compounds), and dietary fibre. In comparison to non-fermented freeze-dried products, fermented snacks had higher levels of 5-methyltryptamine, minerals, 5-methyltryptamine, lowastatin, 6-methyl-D,L-tryptophan, usually antioxidants (phenolic compounds), dietary fibre, and chitin. Additionally, the products from mushrooms fermented with added probiotic cultures *L. acidophilus* or *L. plantarum* exhibited higher levels of indigenous lactobacilli. However, after storage, only microwave-blanched products fermented with *L. plantarum* SWA016 retained high levels of this bacterium. Additionally, these products also contained higher levels of phenolic compounds. The amount of lovastatin and 6-methyl-D, L-tryptophan was highest in water-blanched samples, and 5-hydroxy-L-tryptophan was most abundant in microwave-blanched snacks.

To optimise the content of specific bioactive compounds, the combination of pre-treatment and probiotic strain should be carefully selected. Microwave blanching is more effective in enhancing the levels of K, S, Rb, Fe, Se, Mn, Br, phenolic compounds, antioxidant activity, and soluble dietary fibre. Conversely, water blanching may be preferred for maximising lovastatin content. Since the effect of blanching outweighs that of the added probiotic bacteria on the analysed bioactive compounds, exploring fermentation with alternative probiotic strains may further elucidate their effects on different functional compounds.

## Figures and Tables

**Table 1:** The amount of LAB in fermented and freeze-dried mushrooms (log cfu/g).

		WB	WB + LbA	MB	MB + LbP
After fermentation	Lactococci	7.2 ± 0.1 ^a^	7.2 ± 0.1 ^a^	8.0 ± 0.1 ^b^	8.0 ± 0.1 ^b^
	Lactobacilli	0.0 ^a^	8.2 ± 0.4 ^c^	7.6 ± 0.1 ^b^	8.2 ± 0.2 ^c^
Lyophilised (6 months storage)	Lactococci	6.2 ± 0.3 ^b^	5.5 ± 0.0 ^a^	6.3 ± 0.3 ^b^	6.3 ± 0.1 ^b^
	Lactobacilli	0.0 ^a^	0.0 ^a^	0.0 ^a^	7.3 ± 0.1 ^b^

Legend: means with different small letters represent significant differences at *p* < 0.05, WB—blanched in water and fermented, WB + LbA—blanched in water and fermented with addition of *L. acidophilus* strain LA5, MB–blanched in microwave, MB + LbP—blanched in microwave and fermented with addition of *L. plantarum* strain SWA016.

**Table 2:** Water activity and organic compounds of freeze-dried *L. deliciosus* mushrooms after 6 months of storage.

	NB	WB	WB + LbA	MB	MB + LbP
Water activity	0.24 ± 0.00 ^b^	0.22 ± 0.01 ^a^	0.24 ± 0.01 ^b^	0.27 ± 0.0 ^c^	0.26 ± 0.01 ^c^
Ergothioneine (mg/100 g dm)	35.92 ± 2.30 ^b^	14.86 ± 1.15 ^a^	13.33 ± 1.56 ^a^	36.81 ± 2.30 ^b^	11.81 ± 0.67 ^a^
5-methyltryptamine (mg/100 g dm)	18.89 ± 0.75 ^a^	40.82 ± 2.39 ^c^	27.63 ± 1.41 ^b^	53.39 ± 3.78 ^d^	45.67 ± 4.11 ^c^
Lovastatin (mg/100 g dm)	10.04 ± 0.14 ^a^	23.60 ± 0.63 ^e^	22.47 ± 0.11 ^d^	12.81 ± 0.10 ^c^	10.69 ± 0.18 ^b^
*p*-hydroxybenzoic acid (mg/100 g dm)	1.66 ± 0.15 ^c^	1.31 ± 0.09 ^b^	1.33 ± 0.10 ^b^	0.81 ± 0.04 ^a^	0.73 ± 0.13 ^a^
L-phenylalanine (mg/100 g dm)	*	*	*	*	*
*Indole compounds*					
L-tryptophan (mg/100 g dw)	7.77 ± 0.32 ^d^	3.15 ± 0.08 ^b^	2.27 ± 0.08 ^a^	4.51 ± 0.19 ^c^	3.10 ± 0.20 ^b^
6-methyl-D,L-tryptophan (mg/100 g dm)	3.89 ± 0.19 ^ab^	4.81 ± 0.18 ^ab^	6.76 ± 0.24 ^b^	4.20 ± 0.16 ^ab^	2.70 ± 0.24 ^a^
5-hydroxy-L-tryptophan (mg/100 g dm)	0.65 ± 0.05 ^a^	0.45 ± 0.05 ^a^	0.45 ± 0.05 ^a^	10.85 ± 0.15 ^c^	7.70 ± 0.20 ^b^
Serotonin (mg/100 g dm)	nd	nd	nd	nd	nd

Legend: means with different small letters represent significant differences at *p* < 0.05, NB—fresh, not-blanched and not-fermented, WB—blanched in water and fermented, WB + LbA—blanched in water and fermented with addition of *L. acidophilus* strain LA5, MB–blanched in microwave, MB + LbP—blanched in microwave and fermented with addition of *L. plantarum* strain SWA016, nd—not detected, * present in trace amounts, dm—dry matter.

**Table 3:** Minerals content of fermented freeze-dried *L. deliciosus* after 6 months of storage (mg/100 g dm).

	NB	WB	WB + LbA	MB	MB + LbP
K	11,829 ± 1529 ^b^	8893 ± 1010 ^a^	9004 ± 4475 ^a^	15,774 ± 843 ^c^	17,305 ± 1243 ^c^
Ca	1295 ± 23 ^b^	1243 ± 64 d ^b^	965 ± 522 ^a^	820 ± 54 ^a^	1004 ± 250 ^a^
S	1042 ± 102 ^b c^	935 ± 85 ^ab^	856 ± 440 ^a^	1166 ± 108 ^c^	1008 ± 69 ^ab^
Cl	492 ± 66 ^bc^	428 ± 64 ^b^	285 ± 132 ^a^	548 ± 45 ^c^	413 ± 15 ^b^
Ga	138 ± 0 ^c^	140 ± 0 ^c^	111 ± 0 ^a^	139 ± 0 ^c^	134 ± 0 ^b^
Zn	93.1 ± 1.9 ^c^	89.3 ± 1.8 ^b^	73.6 ± 0.6 ^a^	88.8 ± 0.8 ^b^	102.7 ± 1.0 ^d^
Rb	69.9 ± 5.1 ^b^	64.6 ± 3.7 ^ab^	59.4 ± 1.9 ^a^	100.2 ± 2.5 ^c^	100.3 ± 5.9 ^c^
Fe	50.1 ± 2.8 ^a^	48.2 ± 1.4 ^a^	86.2 ± 0.5 ^c^	66.1 ± 2.8 ^b^	83.8 ± 1.3 ^c^
Cu	33.6 ± 0.6^e^	10.9 ± 0.3 ^a^	15.5 ± 0.1 ^c^	14.9 ± 0.0 ^b^	21.7 ± 0.2 ^d^
Ni	7.56 ± 0.68 ^b^	4.31 ± 0.53 ^a^	16.84 ± 0.27 ^c^	4.74 ± 0.47 ^a^	16.33 ± 0.69 ^c^
Sr	5.41 ± 0.35 ^b^	4.89 ± 0.14 ^b^	3.75 ± 0.38 ^a^	3.69 ± 0.32 ^a^	4.27 ± 0.40 ^a^
Se	2.22 ± 0.04 ^a^	2.29 ± 0.10 ^a^	2.47 ± 0.06 ^b^	2.59 ± 0.06 ^b^	2.95 ± 0.16 ^c^
Mn	2.18 ± 0.11 ^a^	5.20 ± 0.11 ^c^	4.42 ± 0.11 ^b^	6.15 ± 0.24 ^d^	9.00 ± 0.19 ^e^
Br	2.09 ± 0.15 ^c^	1.39 ± 0.14 ^b^	0.75 ± 0.03 ^a^	2.94 ± 0.13 ^d^	2.10 ± 0.26 ^c^
Cr	1.47 ± 0.30 ^a^	1.25 ± 0.12 ^a^	1.55 ± 0.63 ^a^	1.82 ± 0.68 ^a^	1.64 ± 0.70 ^a^

Legend: means with different small letters represent significant differences at *p* < 0.05, NB—fresh, not-blanched and not-fermented, WB—blanched in water and fermented, WB + LbA—blanched in water and fermented with addition of *L. acidophilus* strain LA5, MB–blanched in microwave, MB + LbP—blanched in microwave and fermented with addition of *L. plantarum* strain SWA016.

**Table 4:** Antioxidant activity and phenols content of fermented freeze-dried *L. deliciosus* after 6 months of storage.

	NB	WB	WB + LbA	MB	MB + LbP
Total phenolics [mg chlorogenic acid/g dm]	2154 ± 26 ^b^	1815 ± 44 ^a^	2414 ± 63 ^c^	2529 ± 20 ^d^	2663 ± 49 ^e^
Total tartaric esters [mg caffeic acid/g dm]	440 ± 16 ^b^	391 ± 8 ^a^	559 ± 21 ^c^	614 ± 19 ^d^	642 ± 8 ^e^
Total flavonols [mg quercetin/g dm]	589 ± 11 ^b^	483 ± 9 ^a^	772 ± 13 ^c^	787 ± 19^c^	862 ± 15 ^d^
Total anthocyanins [mg cyanidin/100 g dm]	242 ± 6 ^b^	191 ± 14 ^a^	281 ± 13 ^c^	269 ± 14 ^c^	339 ± 11 ^d^
Lycopene [mg/100 g dm]	4.11 ± 0.03 ^e^	3.48 ± 0.08 ^d^	2.00 ± 0.01 ^a^	2.86 ± 0.12 ^c^	2.66 ± 0.16 ^b^
β-carotene [mg/100 g dm]	8.65 ± 0.13 ^d^	3.92 ± 0.22 ^c^	2.58 ± 0.07 ^a^	2.82 ± 0.18 ^ab^	3.10 ± 0.25 ^b^
DPPH [mg TE/1 g dm]	99.6 ± 9.5 ^b^	68.8 ± 0.9 ^a^	119.2 ± 3.6 ^c^	149.5 ± 3.3 ^d^	142.5 ± 9.5 ^d^
ABTS [mg TE/1 g dm]	494 ± 1 ^b^	350 ± 3 ^a^	510 ± 7 ^c^	705 ± 3 ^e^	572 ± 11 ^d^
FRAP [mM Fe^2+ /^1 g dm]	3893 ± 260 ^a^	4058 ± 146 ^a^	4707 ± 210 ^b^	5876 ± 99 ^c^	4926 ± 285 ^b^

Legend: means with different small letters represent significant differences at *p* < 0.05, NB—fresh, not-blanched and not-fermented, WB—blanched in water and fermented, WB + LbA—blanched in water and fermented with addition of *L. acidophilus* strain LA5, MB–blanched in microwave, MB + LbP—blanched in microwave and fermented with addition of *L. plantarum* strain SWA016.

**Table 5:** Dietary fibre, glucans, chitin, and chitosan of fermented freeze-dried *L. deliciosus* after 6 months of storage (g/100 g dw).

	NB	WB	WB + LbA	MB	MB + LbP
TDF	46.80 ± 1.39 ^c^	50.38 ± 1.54 ^ab^	51.83 ± 1.59 ^b^	48.67 ± 0.99 ^ac^	50.66 ± 1.50 ^ab^
SDF	4.65 ± 0.22 ^a^	4.82 ± 0.19 ^a^	5.12 ± 0.30 ^ab^	5.43 ± 0.50 ^b^	5.21 ± 0.11 ^ab^
IDF	42.15 ± 1.17 ^c^	45.56 ± 1.63 ^ab^	46.71 ± 1.62 ^b^	43.24 ± 1.49 ^ac^	45.45 ± 1.57 ^ab^
Total glucans	26.11 ± 1.11 ^a^	23.12 ± 0.61 ^b^	25.85 ± 0.64 ^a^	19.44 ± 0.43 ^c^	24.53 ± 1.56 ^ab^
α-glucans	0.85 ± 0.03 ^ab^	0.83 ± 0.09 ^a^	0.66 ± 0.03 ^c^	0.81 ± 0.07 ^a^	0.94 ± 0.04 ^b^
β-glucans	25.27 ± 1.09 ^a^	22.30 ± 0.52 ^b^	25.19 ± 0.65 ^a^	18.63 ± 0.37 ^c^	23.59 ± 1.52 ^ab^
Chitin	20.0 ± 1.1 ^c^	21.4 ± 0.6 ^ac^	23.0 ± 0.7 ^ab^	23.1 ± 0.4 ^b^	23.0 ± 0.5 ^ab^
Chitosan	0.64 ± 0.02 ^e^	0.20 ± 0.04 ^ab^	0.15 ± 0.03 ^a^	0.41 ± 0.06 ^d^	0.25 ± 0.05 ^bc^

Legend: means with different small letters represent significant differences at *p* < 0.05, NB—fresh, not-blanched, and not-fermented, WB—blanched in water and fermented, WB + LbA—blanched in water and fermented with addition of *L. acidophilus* strain LA5, MB—blanched in microwave, MB + LbP—blanched in microwave and fermented with addition of *L. plantarum* strain SWA016.

## Data Availability

The original contributions presented in this study are included in the article. Further inquiries can be directed to the corresponding authors.

## References

[1] Fernandes Â., Oliveira B., Martins A., Ferreira I.C. Add–value of *Lactarius deliciosus* and *Macrolepiota procera* wild mushrooms due to their nutritional and nutraceutical potential. Proceedings of the International Congress on Promotion of Traditional Food Products.

[2] Kosanić M., Ranković B., Rančić A., Stanojković T. (2016). Evaluation of metal concentration and antioxidant, antimicrobial, and anticancer potentials of two edible mushrooms *Lactarius deliciosus* and *Macrolepiota procera*. J. Food Drug Anal..

[3] López A.R., Barea–Sepúlveda M., Barbero G.F., Ferreiro–González M., López–Castillo J.G., Palma M., Espada–Bellido E. (2022). Essential mineral content (Fe, Mg, P, Mn, K, Ca, and Na) in five wild edible species of *Lactarius* mushrooms from southern Spain and northern Morocco: Reference to daily intake. J. Fungi.

[4] Tel G., Çavdar H., Deveci E., Öztürk M., Duru M.E., Turkoğlu A. (2014). Minerals and metals in mushroom species in Anatolia. Food Add. Contam. Part B Surveill..

[5] Waktola G., Temesgen T. (2018). Application of mushroom as food and medicine. Adv. Biotech. Microbiol..

[6] Woldegiorgis A.Z., Abate D., Haki G.D., Ziegler G.R. (2015). Major, minor and toxic minerals and anti–nutrients composition in edible mushrooms collected from Ethiopia. J. Food Proc. Technol..

[7] Xu Z., Fu L., Feng S., Yuan M., Huang Y., Liao J., Zhou L., Yang H., Ding C. (2019). Chemical composition, antioxidant and antihyperglycemic activities of the wild *Lactarius deliciosus* from China. Molecules.

[8] Wasser S.P. (2011). Current findings, future trends, and unsolved problems in studies of medicinal mushrooms. Appl. Microbiol. Biotechnol..

[9] Avci E., Avci G.A. (2019). Antimicrobial and antioxidant activities of medicinally important *Lactarius deliciosus*. Int. J. Med. Sci. Dental Res..

[10] Barros L., Baptista P., Estevinho L.M., Ferreira I.C. (2007). Effect of fruiting body maturity stage on chemical composition and antimicrobial activity of *Lactarius* sp. mushrooms. J. Agric. Food Chem..

[11] Ding X., Hou Y., Hou W. (2012). Structure feature and antitumor activity of a novel polysaccharide isolated from *Lactarius deliciosus* Gray. Carbohyd. Polym..

[12] Pogoń K., Jaworska G., Duda–Chodak A., Maciejaszek I. (2013). Influence of the culinary treatment on the quality of *Lactarius deliciosus*. Foods.

[13] Volcão L.M., Halicki P.C.B., Christ–Ribeiro A., Ramos D.F., Badiale–Furlong E., Andreazza R., Bernardi E., da Silva Júnior F.M.R. (2022). Mushroom extract of *Lactarius deliciosus* (L.) Sf. Gray as biopesticide: Antifungal activity and toxicological analysis. J. Toxicol. Environ. Health Part A.

[14] Jonathan G.S., Omotayo O.O., Baysah G.I., Asemoloye M.D., Aina D.A. (2018). Effects of some preservation methods on the nutrient and mineral compositions of three selected edible mushrooms. J. Microb. Biochem. Techn..

[15] Kibar B. (2021). Influence of different drying methods and cold storage treatments on the postharvest quality and nutritional properties of *P. ostreatus* mushroom. Turkish J. Agric. Forest..

[16] Jabłońska-Ryś E., Skrzypczak K., Sławińska A., Radzki W., Gustaw W. (2019). Lactic acid fermentation of edible mushrooms: Tradition, technology, current state of research: A review. Compreh. Rev. Food Sci. Food Saf..

[17] Venugopal K., Satora P., Bernaś E. (2024). Evaluation of sensory and functional compounds in fermented *Lactarius deliciosus* mushrooms. J. Food Proc. Preserv..

[18] Bartkiene E., Zarovaite P., Starkute V., Mockus E., Zokaityte E., Zokaityte G., Rocha J.M., Ruibys R., Klupsaite D. (2023). Changes in lacto–fermented *Agaricus bisporus* (white and brown varieties) mushroom characteristics, including biogenic amine and volatile compound formation. Foods.

[19] Xue Z., Hao J., Yu W., Kou X. (2017). Effects of processing and storage preservation technologies on nutritional quality and biological activities of edible fungi: A review. J. Food Proc. Eng..

[20] Jang H.J., Lee N.K., Paik H.D. (2024). A narrative review on the advance of probiotics to metabiotics. J. Microb. Biotech..

[21] Casertano M., Fogliano V., Ercolini D. (2022). Psychobiotics, gut microbiota and fermented foods can help preserving mental health. Food Re. Int..

[22] Rocks T., West M., Hockey M., Aslam H., Lane M., Loughman A., Jacka F.N., Ruusunen A. (2021). Possible use of fermented foods in rehabilitation of anorexia nervosa: The gut microbiota as a modulator. Progr. Neuro-Psychopharm. Biol. Psych..

[23] Roselli M., Natella F., Zinno P., Guantario B., Canali R., Schifano E., De Angelis M., Nikoloudaki O., Gobbetti M., Perozzi G. (2021). Colonization ability and impact on human gut microbiota of foodborne microbes from traditional or probiotic–added fermented foods: A systematic review. Front. Nutri..

[24] Stiemsma L.T., Nakamura R.E., Nguyen J.G., Michels K.B. (2020). Does consumption of fermented foods modify the human gut microbiota?. J. Nutr..

[25] Taylor B.C., Lejzerowicz F., Poirel M., Shaffer J.P., Jiang L., Aksenov A., Litwin N., Humphrey G., Martino C., Miller–Montgomery S. (2020). Consumption of fermented foods is associated with systematic differences in the gut microbiome and metabolome. Msystems.

[26] Bernaś E., Jaworska G. (2015). Effect of microwave blanching on the quality of frozen *Agaricus bisporus*. Food Sci. Technol. Int..

[27] Zimmer C., Dorea C. (2019). Enumeration of *Escherichia coli* in probiotic products. Microorganisms.

[28] Yang J., Sun Y., Gao T., Wu Y., Sun H., Zhu Q., Liu C., Zhou C., Han Y., Tao Y. (2022). Fermentation and storage characteristics of “Fuji” apple juice using Lactobacillus acidophilus, Lactobacillus casei and Lactobacillus plantarum: Microbial growth, metabolism of bioactives and in vitro bioactivities. Front. Nutri..

[29] Bodzen A., Jossier A., Dupont S., Mousset P.Y., Beney L., Lafay S., Gervais P. (2021). Design of a new lyoprotectant increasing freeze-dried Lactobacillus strain survival to long-term storage. BMC Biotechn..

[30] Basaran D., Fadime Y., Fahrettin G. (2002). Antimicrobial activity of some *Lactarius* species. Pharmac. Biol..

[31] Khalloufi S., Giasson J., Ratti C. (2000). Water activity of freeze dried mushrooms and berries. Can. Agric. Eng..

[32] Li J., Chinachoti P., Wang D., Hallberg L.M., Sun X.S. (2008). Thermal properties of ration components as affected by moisture content and water activity during freezing. J. Food Sci..

[33] Bernaś E., Jaworska G. (2007). Wpływ zabiegów technologicznych na jakość mrożonek z grzybów jadalnych [The impact of technological treatments on the quality of frozen edible mushrooms]. Przem. Ferm. Owoc. Warz..

[34] Chen S.Y., Ho K.J., Hsieh Y.J., Wang L.T., Mau J.L. (2012). Contents of lovastatin, γ–aminobutyric acid and ergothioneine in mushroom fruiting bodies and mycelia. LWT.

[35] Fijałkowska A., Jędrejko K., Sułkowska–Ziaja K., Ziaja M., Kała K., Muszyńska B. (2022). Edible mushrooms as a potential component of dietary interventions for major depressive disorder. Foods.

[36] Muszyńska B., Sułkowska–Ziaja K. (2012). Analysis of indole compounds in edible Basidiomycota species after thermal processing. Food Chem..

[37] Muszyńska B., Sułkowska–Ziaja K., Ekiert H. (2011). Indole compounds in fruiting bodies of some edible Basidiomycota species. Food Chem..

[38] Muszyńska B., Sułkowska–Ziaja K., Wojcik A. (2013). Levels of physiologically active indole derivatives in the fruiting bodies of some edible mushrooms (Basidiomycota) before and after thermal processing. Mycoscience.

[39] Ramakrishnan M., Bey C., Tulasi V., Kislai P., Manohar N. (2017). Investigation of lovastatin, the anti–hypercholesterolemia drug molecule from three oyster mushroom species. Int. J. Biomed. Clinic. Sci..

[40] Paramithiotis S., Das G., Shin H.S., Patra J.K. (2022). Fate of bioactive compounds during lactic acid fermentation of fruits and vegetables. Foods.

[41] Lee K., Lee H., Choi Y., Kim Y., Jeong H.S., Lee J. (2019). Effect of different cooking methods on the true retention of vitamins, minerals, and bioactive compounds in shiitake mushrooms (*Lentinula edodes*). Food Sci. Technol. Res..

[42] Bamidele O., Fasogbon M., Adebowale O., Adeyanju A. (2017). Effect of blanching time on total phenolic, antioxidant activities and mineral content of selected green leafy vegetables. Curr. J. Appl. Sci. Technol..

[43] De Corcuera J.I.R., Cavalieri R.P., Powers J.R., Dekker M. (2004). Blanching of foods. Encyclopedia of Agricultural, Food and Biological Engineering.

[44] Samtiya M., Aluko R.E., Puniya A.K., Dhewa T. (2021). Enhancing micronutrients bioavailability through fermentation of plant–based foods: A concise review. Fermentation.

[45] Ng Z.X., Tan W.C. (2017). Impact of optimized cooking on the antioxidant activity in edible mushrooms. J. Food Sci. Technol..

[46] Sudha G., Vadivukkarasi S., Shree R.B.I., Lakshmanan P. (2012). Antioxidant activity of various extracts from an edible mushroom *Pleurotus ostratus*. Food Sci. Biotechnol..

[47] Alves M.J., CFR Ferreira I., Dias J., Teixeira V., Martins A., Pintado M. (2013). A review on antifungal activity of mushroom (basidiomycetes) extracts and isolated compounds. Curr. Top. Med. Chem..

[48] Cayan F., Deveci E., Tel-Cayan G., Duru M.E. (2020). Identification and quantification of phenolic acid compounds of twenty-six mushrooms by HPLC–DAD. J. Food Measur. Character..

[49] Dudonne S., Vitrac X., Coutiere P., Woillez M., Mérillon J.M. (2009). Comparative study of antioxidant properties and total phenolic content of 30 plant extracts of industrial interest using DPPH, ABTS, FRAP, SOD, and ORAC assays. J. Agric. Food Chem..

[50] Rickman J.C., Barrett D.M., Bruhn C.M. (2007). Nutritional comparison of fresh, frozen and canned fruits and vegetables. Part 1. Vitamins C and B and phenolic compounds. J. Sci. Food Agric..

[51] Su Z., Xu B. (2024). Chemical compositions and health promoting effects of wild edible mushroom milk-cap (*Lactarius deliciosus*): A review. Food Biosc..

[52] Sharma S., Atri N.S., Kaur M., Verma B. (2017). Nutritional and neutraceutical potential of some wild edible *Russulaceous* mushrooms from North West Himalayas, India. Kavaka.

[53] Asamoa A.A., Essel E.A., Agbenorhevi J.K., Oduro I.N. (2018). Effect of processing methods on the proximate composition, total phenols and antioxidant properties of two mushroom varieties. Amer. J. Food Nutr..

[54] Roncero–Ramos I., Delgado–Andrade C. (2017). The beneficial role of edible mushrooms in human health. Curr. Opinion Food Sci..

[55] Mousavi Z.E., Mousavi S.M., Razavi S.H., Hadinejad M., Emam–Djomeh Z., Mirzapour M. (2013). Effect of fermentation of pomegranate juice by *Lactobacillus plantarum* and *Lactobacillus acidophilus* on the antioxidant activity and metabolism of sugars, organic acids and phenolic compounds. Food Biotechn..

[56] Mirończuk–Chodakowska I., Witkowska A.M. (2020). Evaluation of Polish wild mushrooms as beta–glucan sources. Int. J. Environm. Res. Public Health.

[57] Synytsya A., Mickova K., Jablonsky I., SlUKoVá M., Copikova J. (2008). Mushrooms of genus Pleurotus as a source of dietary fibres and glucans for food supplements. Czech J. Food Sci..

[58] Zhang Y., Zhu J., Zou Y., Ye Z., Guo L., Zheng Q. (2024). InsolNBle dietary fiber from five commercially cultivated edible mushrooms: Structural, physiochemical and functional properties. Food Biosci..

[59] Akter M.S., Ahmed M., Eun J.B. (2016). Effect of blanching and drying temperatures on the physicochemical characteristics, dietary fiber composition and antioxidant–related parameters of dried persimmons peel powder. Int. J. Food Sci. Nutr..

[60] Margareta E., Nyman G.L. (2023). Importance of processing for physico–chemical and physiological properties of dietary fibre. Proc. Nutr. Soc..

[61] Knez E., Kadac–Czapska K., Grembecka M. (2023). Effect of fermentation on the nutritional quality of the selected vegetables and legumes and their health effects. Life.

[62] Lambo A.M., Öste R., Nyman M.E.L. (2005). Dietary fibre in fermented oat and barley β–glucan rich concentrates. Food Chem..

[63] Martín–Cabrejas M.A., Sanfiz B., Vidal A., Mollá E., Esteban R., López–Andréu F.J. (2004). Effect of fermentation and autoclaving on dietary fiber fractions and antinutritional factors of beans (*Phaseolus vulgaris* L.). J. Agric. Food Chem..

[64] Mythili S., Rajeswari N., John Don Bosco S., Kamatchi alias Rajalechumi A. (2021). Impact of blanching treatments on the chemical composition, total dietary fiber, physicochemical, functional, and structural properties of underutilized cauliflower leaves (*Brassica oleracea* var. botrytis). J. Food Proc. Preserv..

[65] Zhao H.M., Guo X.N., Zhu K.X. (2017). Impact of solid state fermentation on nutritional, physical and flavor properties of wheat bran. Food Chem..

[66] Bibi A., Xiong Y., Rajoka M.S.R., Mehwish H.M., Radicetti E., Umair M., Shoukat M., Khan M.K.I., Aadil R.M. (2021). Recent advances in the production of exopolysaccharide (EPS) from *Lactobacillus* spp. and its application in the food industry: A review. Sustainability.

[67] Di Cagno R., De Angelis M., Limitone A., Minervini F., Carnevali P., Corsetti A., Gaenzle M., Ciati R., Gobbetti M. (2006). Glucan and fructan production by sourdough *Weissella cibaria* and *Lactobacillus plantarum*. J. Agric. Food Chem..

[68] Alimi B.A., Pathania S., Wilson J., Duffy B., Frias J.M.C. (2023). Extraction, quantification, characterization, and application in food packaging of chitin and chitosan from mushrooms: A review. Int. J. Biolog. Macromol..

[69] Nitschke J., Altenbach H.J., Malolepszy T., Mölleken H. (2011). A new method for the quantification of chitin and chitosan in edible mushrooms. Carbohyd. Res..

[70] Zargar V., Asghari M., Dashti A. (2015). A review on chitin and chitosan polymers: Structure, chemistry, solubility, derivatives, and applications. ChemBioEng Rev..

[71] Satora P., Skotniczny M., Strnad S., Piechowicz W. (2021). Chemical composition and sensory quality of sauerkraut produced from different cabbage varieties. LWT.

[72] Rząsa-Duran E., Muszyńska B., Szewczyk A., Kała K., Sułkowska-Ziaja K., Piotrowska J., Opoka W., Kryczyk-Poprawa A. (2024). *Ilex paraguariensis* Extracts: A Source of Bioelements and Biologically Active Compounds for Food Supplements. Appl. Sci..

[73] Kała K., Pająk W., Sułkowska–Ziaja K., Krakowska A., Lazur J., Fidurski M., Marzec K., Zięba P., Fijałkowska A., Szewczyk A. (2022). *Hypsizygus marmoreus* as a source of indole compounds and other bioactive sNBstances with health–promoting activities. Molecules.

[74] Pansuriya R.C., Singhal R.S. (2009). Supercritical fluid extraction of lovastatin from the wheat bran obtained after solid–state fermentation. Food Techn. Biotechnol..

[75] Zhou T., Liu Q., Jiang W., Chen N. (2014). A new strategy for quantitative analysis of ergothioneine in fermentation broth by RP–HPLC. Proceedings of the 2012 International Conference on Applied Biotechnology (ICAB 2012).

[76] Lazur J., Kała K., Krakowska A., Sułkowska-Ziaja K., Szewczyk A., Piotrowska J., Rospond B., Fidurski M., Marzec K., Muszyńska B. (2024). Analysis of bioactive sNBstances and essential elements of mycelia and fruiting bodies of *Hericium* spp.. J. Food Comp. Anal..

[77] Molyneux P. (2004). The use of the stable free radical diphenylpicrylhydrazyl (DPPH) for estimating antioxidant activity. Songklanakarin J. Sci. Technol..

[78] Thaipong K., Boonprakob U., Crosby K., Cisneros–Zevallos L., Byrne D.H. (2006). Comparison of ABTS, DPPH, FRAP, and ORAC assays for estimating antioxidant activity from guava fruit extracts. J. Food Comp. Anal..

[79] Benzie I.F., Strain J.J. (1996). The ferric reducing ability of plasma (FRAP) as a measure of “antioxidant power”: The FRAP assay. Analit. Biochem..

[80] Fukumoto L.R., Mazza G. (2000). Assessing antioxidant and prooxidant activities of phenolic compounds. J. Agric. Food Chem..

[81] Nagata M., Yamashita I. (1992). Simple method for simultaneous determination of chlorophyll and carotenoids in tomato fruit. Japan. Soc. Food Sci. Technol..

[82] Synowiecki J., Al–Khateeb N.A.A.Q. (1997). Mycelia of *Mucor rouxii* as a source of chitin and chitosan. Food Chem..

